# Prevalence and trends of vitamin D deficiency among patients undergoing testing at the Maternity and Children Hospital, Dammam, Eastern Province of Saudi Arabia (2023–2025)

**DOI:** 10.3389/fpubh.2026.1800317

**Published:** 2026-04-28

**Authors:** Randa Alratrout, Taghreed Alsenbesi

**Affiliations:** Clinical Laboratory Department, Biochemistry Section, Maternity and Children Hospital, Dammam, Saudi Arabia

**Keywords:** Eastern Province, maternal and child health, prevalence, Saudi Arabia, trends, vitamin D deficiency

## Abstract

**Background:**

Vitamin D deficiency (VDD) remains widespread throughout the Middle East despite abundant sunlight. Saudi Arabia reports some of the highest burdens across age groups, with national surveys and cohort studies documenting very high combined rates of deficiency and insufficiency. Lifestyle factors, including limited sun exposure, indoor living, and low physical activity, are consistently implicated. Although high prevalence has been reported among adolescents and women of reproductive age, vitamin D deficiency affects multiple population groups. Earlier evidence from the Eastern Province reported very low circulating 25-hydroxyvitamin D levels despite its coastal latitude, suggesting that behavioral and cultural factors may outweigh geographic advantages. Recent local data continue to show a substantial burden of suboptimal vitamin D status in clinical populations, with nearly half of tested individuals deficient and only about one-fifth achieving sufficient levels, although modest improvements in vitamin D status have been observed over recent years.

**Methods:**

We conducted a retrospective cross-sectional analysis of all serum 25-hydroxyvitamin D [25(OH)D] results measured by CMIA ARCHITECT (Chemiluminescent Microparticle Immunoassay) extracted from the laboratory information system at the Maternity and Children Hospital in Dammam from 1 January 2023 to 31 August 2025. The study population reflects routine clinical testing and includes a heterogeneous mix of pediatric, adolescent, and adult patients. For each calendar year, one index observation per patient was retained. Concentrations were reported in nanomoles per liter (nmol/L). Vitamin D status categories were prespecified as follows: severe deficiency <17.5 nmol/L, deficiency <50 nmol/L, insufficiency 50–74 nmol/L, and sufficiency ≥75 nmol/L. Prevalence was calculated overall and stratified by age group, sex, and year. Associations between vitamin D status and demographic variables were assessed using chi-square tests, and temporal trends were evaluated across study years. Multivariable logistic regression analysis was performed to examine independent predictors of vitamin D deficiency, including age group, sex, and year. Ethics approval was obtained with a waiver of informed consent due to the retrospective nature of the study.

**Results:**

The analytic cohort comprised 14,369 index measurements and reflects a mixed clinical population undergoing routine vitamin D testing. The mean serum 25(OH)D concentration was 56.5 ± 32.6 nmol/L. Overall, 49.1% of results were classified as deficient (<50 nmol/L), 27.4% as insufficient (50–74 nmol/L), 21.2% as sufficient (≥75 nmol/L), and 2.2% as severely deficient (<17.5 nmol/L). Vitamin D deficiency remained highly prevalent across the cohort. Females demonstrated slightly higher rates of deficiency compared with males (50.7% vs. 48.1%, *p* < 0.001). Across study years, the prevalence of deficiency declined modestly from 51.8% in 2023 to 47.6% in 2024 and 47.7% in 2025, with a corresponding gradual increase in sufficiency (19.2, 22.2, and 22.5%, respectively; *p* < 0.001). Multivariable logistic regression analysis showed significant associations between vitamin D deficiency and age group, sex, and year of testing.

**Conclusion:**

Vitamin D deficiency and insufficiency remain highly prevalent among patients tested in this mixed hospital-based population in the Eastern Province. Nearly half of individuals were deficient and over one quarter were insufficient, while only about one fifth achieved sufficient vitamin D levels. Females showed slightly higher rates of deficiency, and modest improvements in vitamin D status were observed across study years. These findings support the need for continued public health measures including routine counseling on safe sun exposure, appropriate vitamin D supplementation across at-risk groups, and sustained food-fortification and awareness programs. Future research should incorporate additional determinants such as anthropometry, dietary intake, supplement use, sun exposure, and genetic factors to better inform targeted interventions.

## 1 Introduction

Vitamin D is a seco-steroid hormone integral to calcium–phosphate homeostasis and skeletal development (1). It also influences immune, metabolic, and reproductive processes (2). Across the Middle East and North Africa, vitamin D deficiency (VDD) remains a major public health concern despite abundant sunlight (3). Restricted skin exposure, indoor lifestyles, clothing practices, and low dietary intake contribute to low circulating 25(OH)D concentrations, and adiposity may further reduce bioavailable vitamin D through sequestration in adipose tissue (4). Saudi Arabia consistently reports some of the world’s highest deficiency rates (5). National surveys and regional cohorts indicate that large segments of the population fall below commonly used sufficiency thresholds (6). Data on children and adolescents are particularly concerning (7), although vitamin D deficiency affects multiple population groups. National surveys and single-center cohorts report high combined prevalence of VDD and insufficiency, with girls and adolescents disproportionately affected (8). In Riyadh, objective measures of outdoor exposure and physical activity correlate with higher 25(OH)D concentrations, yet these behaviors are not sufficiently widespread to normalize population levels (9). Co-existing micronutrient deficiencies, such as iron deficiency, often cluster with VDD, underscoring shared dietary and lifestyle determinants. Similar patterns are observed among adults and women of reproductive age, with high prevalence of low 25(OH)D linked to modifiable factors (10).

Historically, the Eastern Province has also demonstrated high rates of deficiency (11, 12, 13). A hospital-based study from Dammam and Al-Khobar documented extremely low mean 25(OH)D concentrations despite coastal latitude, highlighting that behavioral factors may outweigh geographic advantages in sun exposure (14). More recent analyses suggest that the trajectory is evolving. A five-year retrospective series from central Saudi Arabia reported declining deficiency through 2020 with a slight increase in 2021. Community data from northern regions indicate somewhat lower contemporary prevalence alongside high awareness and supplement use (15, 16, 17). Collectively, these findings establish Saudi Arabia as a high-burden setting with evolving trends and regional variability (18).

Vitamin D status has lifelong implications. During pregnancy, maternal 25(OH)D levels influence fetal accretion and neonatal vitamin D reserves (19, 20). Severe deficiency can lead to neonatal hypocalcemia and rickets and has been linked, although inconsistently, to adverse pregnancy outcomes (21). Mother–infant studies from Saudi Arabia demonstrate strong correlations between maternal and cord concentrations, emphasizing the importance of antenatal screening and supplementation (22, 23). In early childhood, deficiency during rapid growth may impair bone mass accrual, and during adolescence low 25(OH)D levels often coexist with behaviors that compromise bone health, such as indoor leisure activities and limited physical activity (28–31).

Hospital-based data from maternity and pediatric services therefore provide important clinical insight into populations at high risk (7, 23). Despite extensive national literature, recent detailed data from the Eastern Province, particularly from high-volume public hospitals serving mixed patient populations, remain limited (10, 14, 15). The most commonly cited Eastern Province study predates major fortification and awareness initiatives and was not specific to contemporary clinical populations (5, 14). National pediatric syntheses and central-region time-series provide useful context but cannot replace local surveillance due to regional variability and evolving trends (5, 14, 16). In rapidly growing Dammam, hospital-based monitoring can help characterize current prevalence patterns, demographic differences, and short-term temporal changes under real-world conditions (7, 14, 16).

This study aimed to quantify the prevalence and temporal trends of vitamin D deficiency among patients undergoing testing at the Maternity and Children Hospital in Dammam between 2023 and 2025 (7, 14, 15). Specifically, the objectives were to: (1) estimate vitamin D deficiency and insufficiency by age group and sex; (2) examine year-to-year changes in vitamin D status; and (3) describe these findings within the context of a mixed hospital-based population to inform local screening and supplementation strategies. While prior literature emphasizes specific subgroups such as adolescents and women, the present study reflects a broader mixed clinical population undergoing routine testing.

## 2 Methodology

### 2.1 Study design and setting

We conducted a retrospective observational study using serum 25(OH)D measurements extracted from the laboratory information system of the Maternity and Children Hospital, Dammam, a public tertiary center providing obstetric, neonatal, pediatric, and women’s health services. The study population reflects routine clinical testing and includes a heterogeneous mix of pediatric, adolescent, and adult patients. The study period extended from 1 January 2023 through 31 August 2025. The primary outcome was the prevalence of vitamin D deficiency; secondary outcomes included insufficiency, severe deficiency, and temporal trends across study years. Vitamin D concentrations were reported in nanomoles per liter (nmol/L).

Vitamin D status categories were prespecified using cut-points commonly applied in Saudi literature: severe deficiency <17.5 nmol/L, deficiency <50 nmol/L, insufficiency 50–74 nmol/L, and sufficiency ≥75 nmol/L. These thresholds were applied uniformly across all age groups to allow comparability of prevalence estimates. The study followed the STROBE reporting guidelines for observational studies. Because the objective was to describe real-world prevalence and temporal patterns in a hospital-based population, eligibility was not restricted by clinical indication for testing.

### 2.2 Study population

All patients with at least one serum 25(OH)D measurement processed by the hospital laboratory during the study period were eligible, reflecting a mixed clinical population undergoing routine vitamin D testing. Inclusion criteria were a valid patient identifier, specimen date within the study window, and a quantitative 25(OH)D result. We excluded external reference laboratory results, non-serum sample types, analytically invalid results, and clearly miscoded records. To reduce clustering from repeat testing, we retained one observation per patient per calendar year, defined as the earliest measurement within each year for the primary analysis. Age at specimen date was calculated and categorized into clinically relevant groups: 0–9 years, 10–19 years, 20–39 years, and ≥40 years. Sex was extracted as recorded in the laboratory information system. Records with missing demographic data were included in overall prevalence estimates but excluded from stratified analyses.

### 2.3 Data collection and measurement

Data were extracted from the laboratory information system by trained analysts. Variables included patient identifier, sex, date of birth, specimen date, requesting service, clinician code, and serum 25(OH)D concentration (nmol/L). Additional analytic flags and free-text comments were also recorded when available. Serum 25(OH)D concentrations were measured using a chemiluminescent microparticle immunoassay (CMIA) platform with routine internal and external quality control procedures. No analytical platform changes occurred during the study period. All measurements were reported and stored in nanomoles per liter (nmol/L). Vitamin D status categories were assigned using the predefined thresholds described above. Calendar year was derived from the specimen date for temporal analyses. Data quality checks were performed to identify implausible values, date inconsistencies, and cross-field mismatches; records with implausible concentrations were set to missing and flagged for sensitivity analyses.

### 2.4 Statistical analysis

Statistical analyses were performed using SPSS (IBM SPSS Statistics). Continuous variables were summarized using medians and interquartile ranges, while categorical variables were presented as counts and percentages. The prevalence of severe deficiency, deficiency, insufficiency, and sufficiency was calculated overall and stratified by age group, sex, and year. Associations between vitamin D status and demographic variables were assessed using Chi-square (χ^2^) tests. Descriptive statistics were used to summarize vitamin D concentrations, including mean, median, standard deviation, and percentiles. Temporal differences in vitamin D status across study years were evaluated using chi-square tests. Multivariable logistic regression analysis was performed with vitamin D deficiency as the dependent variable and age group, sex, and year as independent variables. Statistical significance was defined as a two-sided *p*-value <0.05.

### 2.5 Ethical considerations

The protocol was reviewed and approved by the Institutional Review Board of the Maternity and Children Hospital, Dammam. Given the retrospective design and use of routinely collected clinical data, the IRB waived informed consent (No. LB-2025-001). Data were de-identified and stored on secure servers. All procedures conformed to the Declaration of Helsinki and local data-protection policies. The use of aggregated hospital-based data was justified to support service evaluation and public health planning.

## 3 Results

### 3.1 Cohort characteristics

Between 1 January 2023 and 31 August 2025, the laboratory identified 14,369 index 25(OH)D measurements, reflecting a mixed clinical population undergoing routine vitamin D testing. Females accounted for 8,272 (59.8%) of the cohort, while males comprised 5,559 (40.2%). The overall median 25(OH)D concentration was 49.1 nmol/L, within the insufficiency range, with a right-skewed distribution and a large proportion of values below 50 nmol/L. The mean serum 25(OH)D concentration was 56.5 ± 32.6 nmol/L. Severe deficiency (<17.5 nmol/L) was present but relatively uncommon, accounting for approximately 2.2% of results (Table 1).

**Table 1:** Vitamin D status by age and sex.

Group	*N*	Mean ± SD	Severe deficiency (%)	Deficiency (%)	Insufficiency (%)	Sufficiency (%)
Overall	14,369	56.5 ± 32.6	2.2	49.1	27.4	21.2
Female (0–9 y)	1,321	79.5 ± 41.5	1.9	42.5	28.4	27.1
Female (10–19 y)	1,755	47.3 ± 23.3	2.2	51.1	27.1	9.7
Female (20–39 y)	1,873	43.8 ± 24.3	4.2	60.5	26.5	8.8
Female (≥40 y)	2,195	41.3 ± 25.7	4.5	73.5	13.2	8.8
Male (0–9 y)	1,514	78.7 ± 41.8	3.8	20.9	29.9	45.4
Male (10–19 y)	1,755	49.5 ± 22.7	1.1	58.9	28.8	11.2
Male (20–39 y)	1,814	48.3 ± 24.0	4.8	57.5	27.5	10.2
Male (≥40 y)	2,152	44.5 ± 23.7	4.7	55.7	25.3	14.3

Sex-stratified totals exclude records with missing sex information; therefore subgroup totals do not equal the overall sample size.

**Figure 1:**
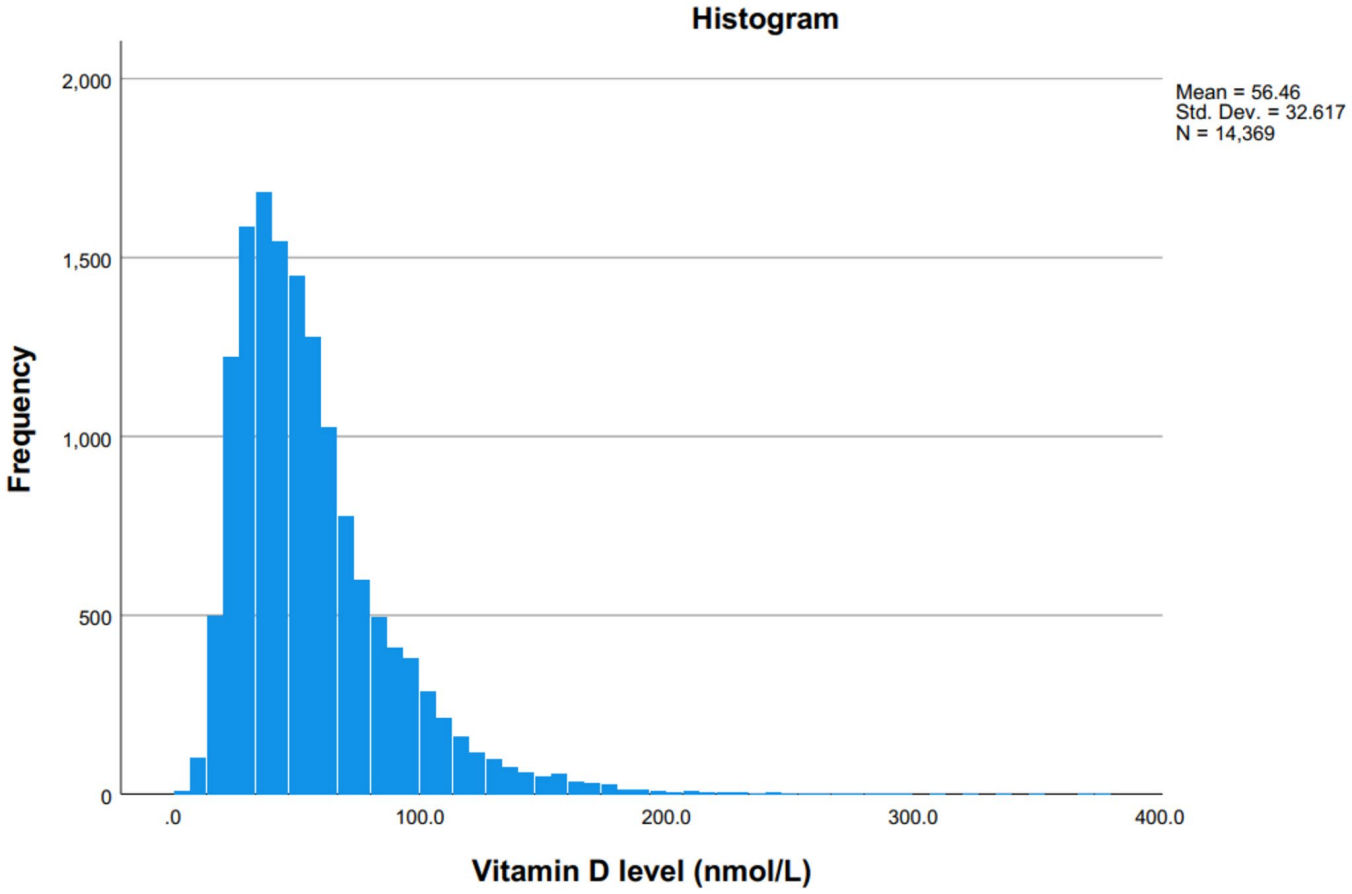
Distribution of serum 25(OH)D concentrations (nmol/L) in the study population.

**Figure 2:**
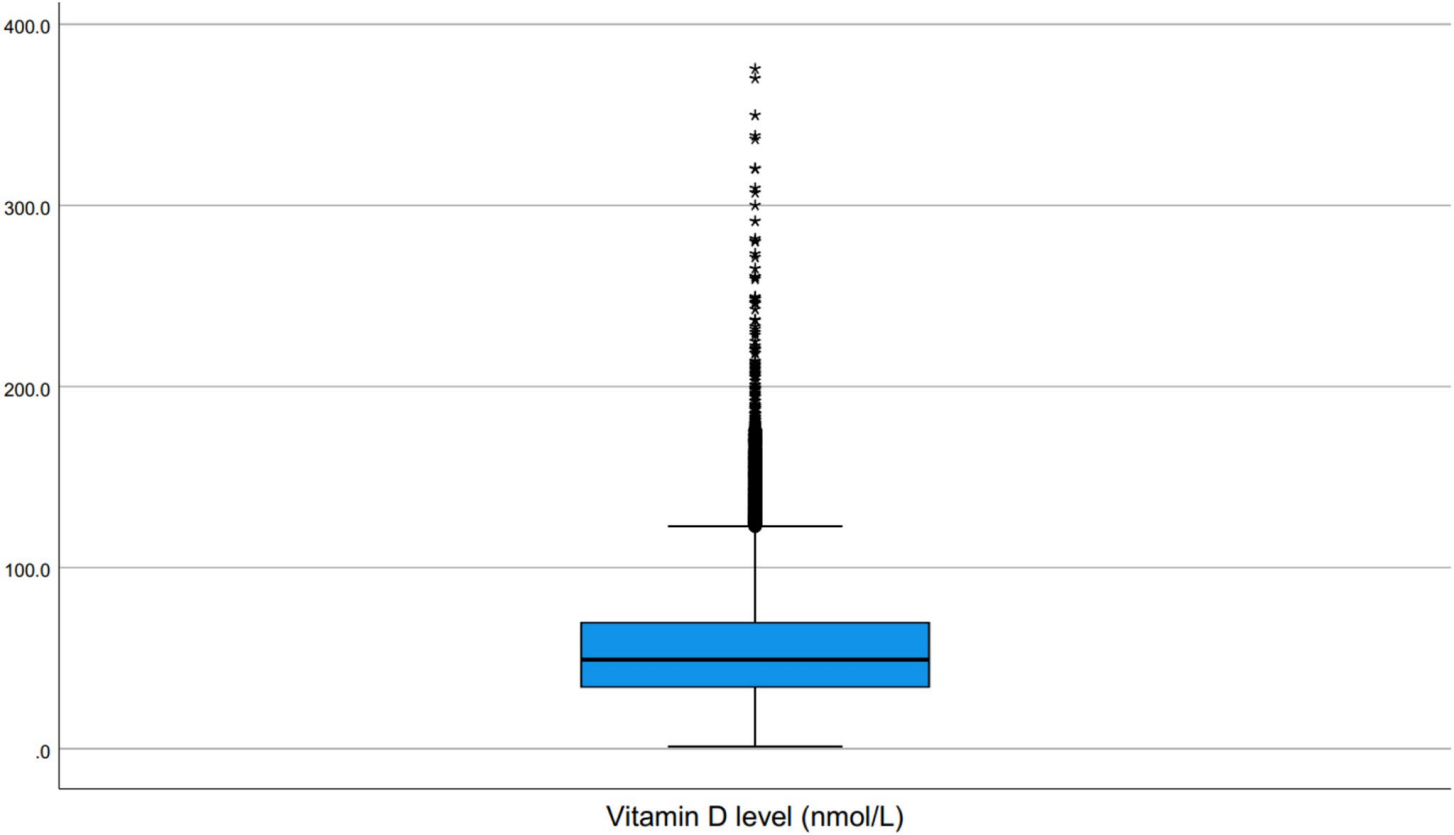
Box plot of serum 25(OH)D concentrations (nmol/L) among patients tested at the Maternity and Children Hospital, Dammam (2023–2025).

The distribution of serum 25(OH)D concentrations across the cohort is illustrated in Figure 1, while Figure 2 presents the distribution of values using a box plot.

### 3.2 Prevalence of deficiency

Vitamin D deficiency was common: 49.1% of index tests were below 50 nmol/L, and a further 27.4% were between 50 and 74 nmol/L. Only 21.2% of results met or exceeded 75 nmol/L. Severe deficiency (<17.5 nmol/L) accounted for approximately 2.2% of measurements. Stratified analyses showed that females had slightly higher deficiency rates than males (50.7% vs. 48.1%). Across the study years, the prevalence of deficiency declined modestly from 51.8% in 2023 to 47.6% in 2024 and 47.7% in 2025, with a corresponding increase in sufficiency. These findings confirm that vitamin D deficiency remains highly prevalent in this hospital-based population in the Eastern Province while indicating modest recent improvements during 2023–2025 (Table 2).

**Table 2:** Descriptive statistics of serum 25(OH)D concentrations (nmol/L) in the study population.

Vitamin D status	*n*	%
Severe deficiency	321	2.2
Deficiency	7,059	49.1
Insufficiency	3,942	27.4
Sufficiency	3,047	21.2

### 3.3 Temporal trends in vitamin D status

#### 3.3.1 Trends over time

Temporal patterns showed modest improvement across the study years. The proportion of results classified as deficient (<50 nmol/L) declined from 51.8% in 2023 to 47.6% in 2024 and 47.7% in 2025. Over the same period, the proportion of sufficient results (≥75 nmol/L) increased from 19.2% in 2023 to 22.2% in 2024 and 22.5% in 2025. Insufficiency (50–74 nmol/L) showed a slight increase, while severe deficiency remained relatively uncommon. These shifts were broadly consistent across demographic groups, suggesting gradual improvement in vitamin D status over time rather than changes limited to a specific subgroup. Overall, the results indicate a modest but measurable improvement in vitamin D status during 2023–2025, while deficiency remains common in this hospital-based population (Table 3).

**Table 3:** Trends in vitamin D status by year (2023–2025).

Year	Severe (%)	Deficiency (%)	Insufficiency (%)	Sufficiency (%)
2023	2.5	51.8	26.4	19.2
2024	2.4	47.6	27.8	22.2
2025	1.6	47.7	28.3	22.5

### 3.4 Subgroup analyses

#### 3.4.1 Age

Vitamin D deficiency varied substantially by age group. Adolescents (10–19 years) showed the highest proportion of results below 50 nmol/L. Children aged 0–9 years had lower but still considerable prevalence, while adults aged 20–39 years showed intermediate levels of deficiency. Adults aged ≥40 years also demonstrated a high prevalence of deficiency. These patterns are consistent with national evidence indicating a substantial burden of vitamin D deficiency across age groups, including adolescents. In younger children, a considerable proportion of results also fell below 50 nmol/L, consistent with studies linking reduced sun exposure and feeding practices to early-life risk (Table 4).

**Table 4:** Distribution of vitamin D status according to age group.

Age group	Severe (%)	Deficiency (%)	Insufficiency (%)	Sufficiency (%)
0–9 y	1.9	42.5	28.4	27.1
10–19 y	2.8	64.3	23.3	9.5
20–39 y	2.8	49.3	28.3	19.6
≥40 y	1.5	42.0	31.5	25.0

#### 3.4.2 Sex

Females exhibited slightly higher prevalence of vitamin D deficiency compared with males. Deficiency (<50 nmol/L) was observed in 50.7% of females compared with 48.1% of males, while sufficiency (≥75 nmol/L) was slightly lower among females (20.2% vs. 21.5%). These findings are consistent with national Saudi studies reporting somewhat higher deficiency rates among females, often attributed to factors such as clothing practices, reduced sun exposure, and lower levels of outdoor physical activity (Table 5).

**Table 5:** Distribution of vitamin D status according to sex.

Sex	Severe (%)	Deficiency (%)	Insufficiency (%)	Sufficiency (%)
Male	1.7	48.1	28.6	21.5
Female	2.7	50.7	26.3	20.2

#### 3.4.3 Service stream

Most vitamin D tests in this cohort were requested for pediatric and adolescent patients, reflecting the age distribution of the hospital-based population undergoing testing. Consistent with this structure, a large proportion of deficient results occurred in younger age groups. Within maternity services, many values fell within the insufficiency range (50–74 nmol/L), while a considerable proportion remained below 50 nmol/L. These findings are broadly consistent with Saudi studies reporting high prevalence of vitamin D deficiency in antenatal populations. Neonatal and early childhood services also demonstrated a broad distribution of vitamin D concentrations, with many values remaining below recommended sufficiency thresholds.

#### 3.4.4 Month

Exploration of temporal patterns across the study period showed modest variation without a clear seasonal peak. The overall distribution of vitamin D concentrations remained relatively stable across months. This pattern may reflect behavioral sun avoidance during periods of extreme heat and the relatively short observation window of approximately 2.7 years.

### 3.5 Multivariable analysis

Multivariable logistic regression analysis demonstrated significant associations between vitamin D deficiency and age group, sex, and year of testing (Table 6).

**Table 6:** Multivariable logistic regression analysis of factors associated with vitamin D deficiency.

Variable	OR (Exp B)	95% CI	*p*-value
Age group (10–19)	2.87	2.49–3.31	<0.001
Age group (20–39)	1.35	1.17–1.57	<0.001
Sex (female)	0.83	0.77–0.90	<0.001
Year (2024)	1.29	1.18–1.41	<0.001
Year (2025)	1.07	0.98–1.17	0.120

## 4 Discussion

### 4.1 Comparison with existing literature

A substantial proportion of patients tested in this hospital-based population had suboptimal vitamin D status. The overall deficiency rate (49.1%) and insufficiency rate (27.4%) place this population within the high-burden range reported in Saudi studies (7, 14, 16). Previous meta-analyses of Saudi populations have estimated that roughly two thirds of adults are vitamin D deficient (4, 8, 14). In the present study, adolescents showed a higher prevalence of deficiency, consistent with national surveys and cohort studies documenting elevated rates in this age group (4, 16). Younger children also showed considerable deficiency, aligning with regional studies linking limited sun exposure and feeding practices to early-life risk (7, 9, 28).

The Eastern Province has historically reported particularly low vitamin D levels (14). Earlier hospital-based research from Dammam and Al-Khobar documented very low mean circulating 25(OH)D concentrations despite the region’s coastal latitude (5). Against this background, our 2023–2025 data show modest improvement over time, with the proportion of deficiency declining from 51.8% in 2023 to 47.6% in 2024 and 47.7% in 2025. Similar gradual improvements have been reported in other Saudi regions, where increasing awareness and supplement use may contribute to improving vitamin D status (16, 17). Within maternity services, our observation that many results fall within the insufficiency or deficiency ranges is broadly consistent with Saudi studies reporting low vitamin D levels in antenatal populations (7, 22).

The absence of a pronounced seasonal pattern in our data is plausible given the desert climate, where extreme heat discourages outdoor activity during much of the year (10, 14). This contrasts with temperate regions where vitamin D levels show clear seasonal variation and further supports the view that behavioral factors, rather than solar irradiance alone, are major determinants of vitamin D status in Saudi Arabia. Although the modest improvement observed over time is encouraging, the majority of patients still fall below sufficiency thresholds, highlighting the ongoing public health burden in this clinical population (9, 14, 17).

### 4.2 Interpretation for pediatric and maternal health

From a pediatric perspective, the high prevalence of deficiency and insufficiency across childhood and adolescence is clinically important. Vitamin D status during childhood influences bone mineralization and may affect long-term skeletal health. In our cohort, adolescents (10–19 years) showed the highest prevalence of deficiency, with more than half of results in this age group falling below 50 nmol/L. These findings are consistent with national evidence that adolescents carry a substantial burden of low vitamin D status. These patterns are often attributed to indoor lifestyles, reduced outdoor physical activity, and cultural practices that limit sun exposure. Younger children also demonstrated considerable deficiency, highlighting the importance of early-life supplementation and caregiver education.

Among adults aged 20–39 years, deficiency remained common, while adults aged ≥40 years also showed a high prevalence of deficiency. Within maternity services, many results fell within the insufficiency or deficiency ranges, broadly consistent with Saudi studies reporting low vitamin D levels in antenatal populations. Although associations between maternal vitamin D status and pregnancy outcomes remain variable across studies, low maternal levels can adversely affect neonatal vitamin D reserves and have been linked to neonatal hypocalcemia and rickets. These findings support continued antenatal counseling and vitamin D supplementation strategies as part of broader clinical care.

### 4.3 Causes and cultural factors

The determinants of vitamin D deficiency in Saudi Arabia are multifactorial. Limited sun exposure due to indoor schooling, urban lifestyles, extreme heat, and clothing practices is considered a major contributor (4, 9). Low intake of vitamin D–rich foods and inconsistent consumption of fortified products may further contribute to persistent insufficiency across different population groups (10). Urban residence and limited opportunities for outdoor activity have also been associated with lower vitamin D levels among children (9, 14). The slightly higher prevalence of deficiency observed among females in our cohort is consistent with national findings and is commonly attributed to factors such as reduced sun exposure, clothing coverage, and lower participation in outdoor physical activity (7, 30, 31). Historical studies from the Eastern Province demonstrate that proximity to coastal areas and high ultraviolet exposure do not necessarily translate into adequate vitamin D status when behavioral and cultural factors limit effective sun exposure (5, 9, 14). Recent reports of modest improvements in vitamin D status in some Saudi regions have been attributed to increased awareness, supplementation, and food fortification programs (14, 17). Community-based studies linking higher awareness to lower prevalence further emphasize the role of public health education in addressing vitamin D deficiency (18).

### 4.4 Strengths and limitations

A major strength of this study is its large contemporary dataset comprising 14,369 index measurements from a high-volume public hospital. The use of prespecified vitamin D status thresholds consistent with Saudi literature enhances comparability with previous studies. Restricting the primary analysis to one observation per patient per calendar year reduced potential bias from repeat testing. In addition, laboratory measurements were performed using a consistent analytical platform throughout the study period, supporting comparability of results over time.

Several limitations should be considered. First, the cohort includes only patients who underwent vitamin D testing, which may overrepresent individuals with clinical indications and therefore potentially overestimate prevalence compared with the general population. Second, important covariates such as body mass index, dietary intake, supplement use, sun exposure, and clothing practices were not available in the laboratory dataset, limiting causal interpretation. Third, the single-center design may limit generalizability beyond the Eastern Province, although the observed patterns are broadly consistent with national data. Fourth, observational data cannot fully distinguish changes in testing patterns from true shifts in population vitamin D status over time. Finally, while standardized thresholds were used to categorize vitamin D status, alternative cut-points exist in the literature and could produce different prevalence estimates.

### 4.5 Public health and clinical implications

The high prevalence of vitamin D deficiency and insufficiency observed in this cohort highlights the need for multi-level public health and clinical interventions (7, 16). Clinicians should provide brief counseling on safe sun exposure appropriate to the local climate and promote standardized vitamin D supplementation across relevant clinical groups, including antenatal and pediatric care pathways where appropriate (9, 22, 32). School-based programs encouraging outdoor activity and nutrition education may also help improve vitamin D status. At the population level, maintaining and monitoring food fortification programs and public-awareness campaigns remain important strategies (16, 22). Evidence from regions with higher awareness and supplement use suggests that education and preventive strategies can reduce deficiency prevalence (16). Health services may also benefit from implementing quality-improvement monitoring systems to track vitamin D status and supplementation practices. Future research should incorporate additional determinants such as body mass index, dietary intake, supplement use, sun exposure, and genetic factors to better refine risk stratification and evaluate the effectiveness of targeted interventions. Multi-center studies and pragmatic trials within the Eastern Province could further inform regional prevention and supplementation policies (33).

## 5 Conclusion

In summary, vitamin D deficiency remains highly prevalent among patients tested in this hospital-based population in the Eastern Province. Nearly half of individuals tested between 2023 and mid-2025 had 25(OH)D concentrations below 50 nmol/L, and more than one quarter were classified as insufficient. Adolescents showed a higher burden of deficiency, and females demonstrated slightly higher prevalence compared with males. Although modest improvement in vitamin D status was observed across the study years, the majority of patients still did not reach sufficiency. These findings support the need for continued interventions including counseling on safe sun exposure, appropriate vitamin D supplementation across relevant groups, promotion of outdoor activity, and sustained public-health awareness campaigns. Future studies should incorporate anthropometric, dietary, behavioral, and genetic factors to better identify high-risk groups and evaluate the impact of targeted interventions.

## Data Availability

The original contributions presented in the study are included in the article/supplementary material, further inquiries can be directed to the corresponding author.
